# Learning to read Chinese: the roles of phonological awareness, paired–associate learning, and phonetic radical awareness

**DOI:** 10.1007/s11145-022-10352-9

**Published:** 2022-10-05

**Authors:** Chien-Chih Tseng, Jon-Fan Hu, Li-Yun Chang, Hsueh-Chih Chen

**Affiliations:** 1 Department of Educational Psychology and Counselling, National Taiwan Normal University, 162 He-ping E. Rd., Sec 1, Taipei, 10610 Taiwan, Republic of China; 2 Department of Psychology, National Cheng Kung University, Tainan, Taiwan; 3 Department of Chinese as a Second Language, National Taiwan Normal University, Taipei, Taiwan; 4 Institute for Research Excellence in Learning Sciences, National Taiwan Normal University, Taipei, Taiwan; 5 Chinese Language and Technology Centre, National Taiwan Normal University, Taipei, Taiwan

**Keywords:** Chinese character, Chinese reading acquisition, Phonological awareness, Paired–associate learning, Phonetic radical awareness

## Abstract

This study aimed to determine how Chinese children adapt to Chinese orthography–phonology correspondence by acquiring phonetic radical awareness (PRA). This study used two important Chinese encoding approaches (rote and orthographic approaches) as the developmental trajectory, in which the present study hypothesized that phonological awareness (PA) exerts not only a direct influence on PRA but also an indirect influence through paired– associate learning (PAL). We also explored whether the association between PA and PAL is affected by the complexity of visual stimuli embedded in PAL. This study recruited 70 s-grade students to participate in various tests, which assessed (a) PA (measured by onset and rhyme awareness), (b) PRA (measured by regularity and consistency of phonetic radicals), (c) PAL (measured by learning performance on strokes; pattern-object and strokes pattern-syllable mapping), and (d) Chinese character recognition ability. Path analyses indicated that (1) character size had a significant positive correlation with PRA but not with PAL, (2) PAL fully mediated the association between PA and PRA, and (3) compared with PAL with a low stroke count, PA had a stronger relationship with PAL with a high stroke count. The results of this study were consistent with previous studies and suggest that PRA is the most important literacy skill for children in the middle of their learning-to-read stage. The results also augment existing literature by revealing that PRA acquisition is increased by PAL supported by PA, rather than by PA alone. Moreover, when the visual complexity of PAL increases, the support of PA to PAL would increase to make up for the working memory shortage.

## Introduction

Paired–associate learning (PAL) and phonological awareness (PA) are the basic cross-language abilities employed by beginning readers to acquire subsequent reading skills (e.g., Ehm et al., 2019; Georgiou et al., 2017; Hulme et al., 2007; Liu et al., 2021). The acquisition of reading ability is a continuous process of understanding how visual symbols map to familiar oral speech (Liu et al., 2021b). PAL refers to the ability to create stimulus–response connections in memory (Georgiou et al., 2017), whereas PA comprises the ability to recognize, identify, or manipulate any phonological unit within a word (Ziegler & Goswami, 2005). Both PAL and PA play prominent roles in phonological recording to retrieve the meaning of words in alphabetic languages (Hulme et al., 2007). While the Chinese writing system lacks letter(s)–sound correspondence, there is robust evidence (Ho & Bryant, 1997a; Ho et al., 2003a; Li et al., 2020; Shu et al., 2000; Tzeng et al., 1995) that Chinese characters could still be recognized through phonetic radical awareness (PRA). PA and PAL are known to make independent contributions to word reading during the development of Chinese reading ability (Chow, 2014; Georgiou et al., 2017; Liu et al., 2021b), but how PA and PAL apply to Chinese orthography to acquire PRA is still unclear.

PRA formation in Chinese is strongly influenced by literacy experience (Chan & Wang, 2003; Tzeng & Lee, 2012), which beginning readers can enhance by using PA and PAL to facilitate arbitrary associations between forms, sounds, and meanings of new characters (Georgiou et al., 2017; Liu et al., 2021b). This study therefore applied the rote-learning (i.e., encoding novel characters by rote at a holistic level) and orthographic (i.e., encoding novel characters using knowledge of radical functions) approaches to construct a hypothesized mediation model. In that model, PAL and PRA were assumed to be the core abilities in the rote-learning and orthographic approaches, respectively, and PAL was assumed to mediate the association between PA and PRA.

Visual complexity is higher for Chinese characters than in alphabetic orthographies (Chang et al., 2018, 2022). This study therefore further distinguished types of PAL with different visual complexities, and explored how regression coefficients between PA and PAL are affected by the number of strokes embedded in PAL based on our assumption that PA provides more support under the restricted attentional resources induced by complex visual coding.

Since most previous studies have investigated alphabetic languages, the present study aimed to determine (1) how PAL interacts with PA to enhance PRA, which is a fundamental Chinese literacy skill, and (2) whether the strength of the association between PAL and PA is affected by the visual complexity of the glyph. Before we review the literature related to how PA and PAL contribute to PRA in Chinese, we first describe Chinese orthography characteristics.

### Characteristics of Chinese orthography

Chinese is a nonalphabetic language in which the basic unit, the character, represents a syllable and a morpheme. The morphosyllabic nature of Chinese characters makes written Chinese a unique writing system (Daniels & Bright, 1996). Moreover, a single Chinese character is on average five times more complex than a single English letter (Chang et al., 2018). The complex Chinese writing system has three levels: characters, radicals, and strokes. Radicals are the building blocks of characters and comprise strokes (Tseng et al., 2018). Most radicals are standalone Chinese characters (known as sinograms), while few radicals are bounded. An analysis of 6097 frequently used characters (Chen et al., 2011) found that 1.2% were sinograms and 98.8% were compounds. Semantic-phonetic compounds account for 80–90% of Chinese characters (Li & Kang, 1993; Rogers & Rogers, 2005), and consist of a semantic radical (indicating the meaning of the entire character) and a phonetic radical (indicating how to pronounce it). For example, in the semantic-phonetic compound  (pronounced as fēng, and meaning “maple”),  (meaning “wood” as a sinogram) is the semantic radical whose meaning is related to that of the entire character, while  is the phonetic radical and has the same pronunciation as the entire character.

Empirical data support that knowledge of radical functions facilitates the learning of new characters (e.g., Li et al., 2020, 2021), but the phonetic information from radicals is quite unreliable. According to character corpus statistics, 33.5% of semantic-phonetic compounds have the same onset and rhyme as their phonetic radicals when excluding lexical tone information (Hsiao & Shillcock, 2006), whereas 36% have a meaning related to their semantic radical (Li & Su, 2011). Given the low phonetic regularity of Chinese characters, acquiring an understanding of Chinese characters would therefore rely partly on external teaching and environmental language experiences.

### Developmental trajectory in reading Chinese: from the rote-learning to the orthographic approach

When learning alphabetic languages, young children might initially read logographically (Frith, 1985) and identify words by remembering the salient visual features of symbols, without understanding that a certain letter represents a specific sound. Formal teaching will allow children to master more grapheme-to-phoneme correspondences in order to convert letters (grapheme sequence) into their pronunciations (phoneme sequence), and then obtain the meaning.

Chinese reading ability also has a similar general developmental trajectory from a rote-learning to an orthographic approach. Many previous studies have supported this developmental framework for the acquisition of Chinese reading ability (Chow, 2018a, 2018b; Siok & Fletcher, 2001). For example, Chow (2018b) investigated the analytic strategy that children apply when learning Chinese characters at different developmental stages. Students at different developmental stages (kindergarten, second grade, and fifth grade) were asked to learn regular and irregular pseudocharacters, and it was found that the influence of regularity on pseudocharacter learning varied with age. The results supported a stage-like model in which beginning readers rely on a holistic approach, which gradually morphs into an analytic approach to word learning as they have more literacy experiences. Based on these previous findings, the present study proposed a developmental trajectory for Chinese reading ability from a rote-learning to an orthographic approach to explore and expand the understanding of the roles of PA and PAL in Chinese reading development.

Chinese characters are considered as logographic scripts. Beginning Chinese readers acquire visual-based configuration knowledge of Chinese characters (Ho et al., 2003b; McBride, 2004), but are not yet able to analyze and apply the knowledge of radicals contained within novel characters. Instead, children holistically memorize both form–sound and shape–meaning connections by rote, a process that is heavily dependent on PAL. PAL should therefore be a critical ability for the rote-learning approach.

Character size (CS) refers to the number of characters that a student approximately knows, and represents a basic decoding skill for Chinese reading comprehension, similar to the vocabulary size in alphabetic languages. However, Chinese readers take years to master the 2328 characters that are commonly used in everyday life (Huang, 1994). Rote-learning readers learn each character independently, which can lead to memory overload (Ho & Bryant, 1997a). To reduce the cognitive load when learning Chinese characters, Chinese learners will memorize the visual orthographic information (Chang et al., 2014) or extract the phonetic or semantic regularities of the radicals (Shu et al., 2003), which is known as Chinese orthographic awareness (OA). PRA is a critical part of OA in the orthographic approach (Ho et al., 2003a). According to the self-teaching hypothesis (Share, 1995), word-specific orthographic representations are primarily acquired by translating novel letter strings into sounds using phonological recoding. Although Chinese is a nonalphabetic script without phonological recording processing, PRA could still contribute to the acquisition of unfamiliar Chinese characters. For example, Li et al., (2020, 2021) tested the self-teaching hypothesis on Chinese second-grade students, with their results supporting the primary role of phonological recoding in character learning.

Chinese children use two kinds of grapheme–sound conversions to guess the sound of novel characters using phonetic radicals (Hsiao & Shillcock, 2006; Liu et al., 2003): regularity and consistency. Regularity refers to the extent to which a character sound is similar to that of its phonetic radical, whereas consistency refers to the extent to which compounds sharing a common phonetic radical have similar pronunciations. The initial logographic phase may be necessary for gradually developing phonetic regularity and consistency (Ho & Bryant, 1997a) before the mature application of PRA to improve word reading. In phonetic regularity and consistency, children must rely on the Chinese characters they have acquired to make an analogy or to derive based on the phonetic, respectively, to guess the pronunciation of novel characters (Chan & Wang, 2003).

There is empirical evidence that PRA independently contributes to Chinese word reading when other important language skills are controlled (Ho & Bryant, 1997a; Ho et al., 2003a; Tong et al., 2017). Lee et al. (2006) used event-related potentials to examine the time course of sublexical orthography-to-phonology (OTP) transformation when reading pseudocharacters containing phonetic radicals. They found that when reading Chinese pseudocharacters, pseudocharacters paired with unpredictable pronunciations elicited larger P200 (an index of the early extraction of phonology) and N400 (related to post lexical processing) than did those paired with predictable pronunciations. This finding suggests that pseudocharacters—which are novel characters for readers—automatically activate their pronunciations, in addition to the consistency effect occurring during the early stage of lexical processing. Together these findings support the presence of a significant positive association between PRA and CS for school-aged children, but no significant association between PAL and CS.

### PAL contributes to PRA

PAL tasks can involve unimodal (e.g., visual–visual) or cross-modal (e.g., visual–verbal) stimuli. Word reading has been more strongly associated with visual–verbal tasks than with visual–visual and verbal–verbal learning tasks (e.g., Hulme et al., 2007; Messbauer & Jong, 2003; Wang et al., 2017a, 2017b) in alphabetic orthographies. Visual–verbal PAL is a similarly important skill contributing to Chinese reading ability (Georgiou et al., 2017). Regarding unimodal PAL, different from findings in alphabetic orthographies, previous research suggested that the extent to which visual–visual PAL contributes to Chinese reading ability varies with the level of semantic accessibility that the visual stimuli used in PAL involved. Huang and Hanley (1995) found a significant relationship between performance on a visual–visual PAL task (requiring children to learn the color associated with abstract line drawings) and reading ability. Li et al. (2009) showed the ability to connect abstract visual symbols and pictures of a familiar animal with a new feature was linked to Chinese word reading ability. In contrast, Wang and Allen (2018) reported that visual–visual PAL (requiring children to learn the unfamiliar figures associated with abstract shapes) was not significantly associated with word reading when visual–verbal PAL was considered. This could be attributed to the logosyllabic nature of the Chinese writing system resulting in children’s performance on high semantic accessibility visual–visual PAL significantly contributing to lexical development.

PAL is a critical ability for acquiring knowledge about the letter(s)–sound correspondence in alphabetic writing systems, but the extent to which PAL contributes to OTP rules in a nonalphabetic writing system such as Chinese (which is referred to as PRA here) is still unclear. The present study assumed that PAL not only plays a prominent role in the rote-learning approach but also serves as the key function in developing PRA. The PRA development process from beginning to maturity is slow and incremental, and would occur over all elementary-school ages (Tzeng & Lee, 2012). Before sophisticatedly employing radical functions in orthographic learning, beginning learners need to achieve a certain level of literacy using the rote-learning approach (Ho & Bryant, 1997a), which is conducted by PAL.

### PA contributes to PRA through PAL

Previous studies of alphabetic writing systems have supported the close relationship between PA and word reading (e.g., Ziegler, 2005), since good PA helps readers to make a fine-grained phonological segmentation at the phoneme level and convert letter(s) into speech sound(s). However, the contribution of PA to the ability to read Chinese is not only unstable throughout the developmental trajectory, but also unclear because it is associated with other cognitive skills. Specifically, studies involving preschool children (e.g., Ho & Bryant, 1997b; McBride-Chang & Kail, 2002; Shu et al., 2008) have found that PA markedly influences the subsequent performance in word reading. In contrast, the relationship between PA and word reading becomes weaker in elementary-school children when the research models include orthographic knowledge (Liao et al., 2014; Tong et al., 2009), morphological awareness (Liao et al., 2014; McBride-Chang, 2004; Tong et al., 2009), or earlier-grade word reading ability (Yeung et al., 2013). These results collectively suggest that PA is a basic cognitive ability that interacts with other abilities in learning to read Chinese. To address the complex relationship among abilities, instead of using regression (like in previous studies), the present study adopted path analysis. This approach goes beyond multiple regression (Streiner, 2005) to investigate the relationships among PA and various cognitive abilities to elucidate how PA influences the process of learning to read Chinese.

In contrast to previous research of alphabetic orthographies consistently suggesting that PA facilitates a phonological representation in PAL, which in turn enhances reading skills (Ehm et al., 2019; Mourgues et al., 2016), inconsistent results were found for Chinese by Liu et al., 2021; Liu & Chung, 2021). Liu and Chung (2021) found that PAL mediates the relationship between PA (measured using a syllable deletion task) and word reading, after controlling for the PA, orthographic knowledge, visual–motor integration, inhibition, ability to read words, age, and nonverbal intelligence quotient (IQ) of second-year kindergarten children (K2). However, Liu et al. (2021) found no such relationship between PA (also measured using a syllable deletion task) and PAL in third-year kindergarten children (K3). To explain this discrepancy, the present study considered the descriptive statistics of these studies, and found that K3 children seemed to outperform K2 children in a verbal working memory task (mean scores of 8.24 and 5.17, respectively, out of a maximum of 28). It can be presumed that the higher verbal working memory capacity of K3 children diminished the effect of PA on PAL.

Previous research into Chinese reading ability appears to support the effect of PA on PAL, which in turn enhances word reading. However, those studies did not consider the mediating role of PRA in word reading. In the absence of letter(s)–sound correspondence in Chinese, Ho and Bryant (1997a) investigated whether PA (rhyme awareness) is important for learning these script–sound regularities. Their correlation analysis revealed a significant positive correlation between PA and word reading in first-grade students. However, their multiple regression analysis of using IQ, PA, and PRA together to predict word reading simultaneously showed that only PRA was correlated with word reading, while there was no longer a positive correlation between rhyme awareness and word reading. Those authors concluded that the association between rhyme awareness and word reading might be attributable to rhyme awareness promoting the learning of PRA.

To summarize, the results of Ho and Bryant (1997a) suggest that PA can directly affect PRA, but that study did not consider the role of PAL in mediating the relationship between PA and PRA. Furthermore, given that PAL was found to be a partial mediator of the association between PA and word reading in alphabetic (Ehm et al., 2019; Mourgues et al., 2016) and nonalphabetic (Liu et al., 2021) orthographies, the present study hypothesized that PAL partly mediates the association between PA and PRA.

### Effect of visual complexity: different stroke counts embedded in PAL

Chinese is characterized by a high degree of visual complexity, and so this study attempted to determine how the visual complexity of PAL affects the relationship between PA and PAL. The visual complexity of Chinese characters is mostly affected by their constituent strokes. Su and Samuels (2010) found that more processing time was required to recognize characters comprising more strokes among second-grade students, but not among fourth-grade, sixth-grade, or university students.

PAL involves multiple processes: stimulus learning (the visual representation), response learning (the phonological representation), and the formation of a stimulus–response association (Georgiou et al., 2017; Hulme et al., 2007). Establishing and integrating across the phonological and visual representations in memory, embedded in PAL, requires working memory processing (Berninger et al., 2015; Wang & Allen, 2018; Wang et al., 2017a, 2017b). Ehm et al. (2019) consistently found that working memory independently predicted the PAL of German children. Liu et al. (2021) similarly found that working memory is a key capability for PAL support in Chinese children. Different from alphabetical languages, learning to read Chinese requires children to memorize unfamiliar characters with a high degree of visual complexity, and so successful PAL requires attentional resources for encoding visual symbols.

School-aged children have relatively low attentional resources. An individual uses attentional mechanisms to select only a subset of the sensory input to process (Wahn & König, 2017). Successful PAL in Chinese might require considerable attentional resources, since it demands the creation and integration of phonological representations with highly visually complex and unfamiliar representations. Considering the cross-modal nature of shared attentional resources (Engle et al., 1999), children may allocate considerable attentional resources to the complex visual representation and thereby reduce the attentional resources available for phonological representations. Therefore, phonological encoding efficiency is of importance when attentional resources are restricted. We therefore assumed that children with good PA still bind novel phonological representations with another visual symbol in PAL. The present study manipulated the number of strokes in PAL to examine whether PA has a stronger association with high-stroke-count PAL (HSPAL) than with low-stroke-count PAL (LSPAL).

### Research hypotheses

With the overarching goal of identifying the roles of PA and PAL in influencing PRA, the present study proposed a developmental trajectory with two encoding approaches (rote learning and orthographic) to establish and validate a hypothesized model. The model was used to investigate visual complexity embedded in PAL in order to elucidate the association between PA and PAL and their roles in learning to read Chinese, the nonalphabetic writing system. This study analyzed individual differences to explore the relationships among various abilities, and recruited 70 s-grade Chinese students since this is probably the stage with the fastest increase in literacy ability (Wang et al., 2008), thereby maximizing the individual differences regarding CS.

**Fig. 1:**
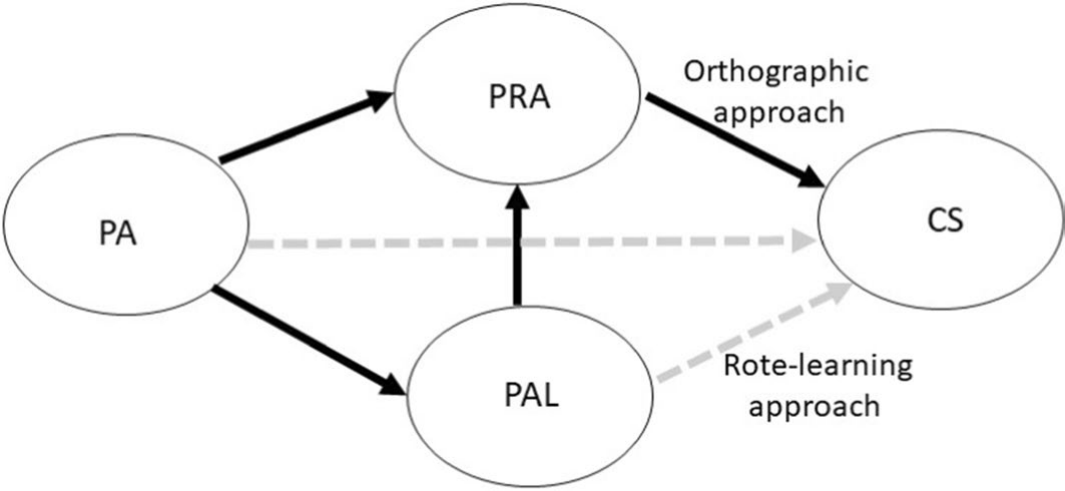
Hypothesized model for predicting character size (CS) using phonological awareness (PA) under two approaches. PRA, phonetic radical awareness; PAL, paired association learning. Note. Solid black lines indicate hypothesized significant paths, and dotted gray lines indicate hypothesized nonsignificant paths

Figure 1 presents the hypothesized model for predicting CS using PA for Hypotheses 1 and 2 (see below). For Hypothesis 1, instead of the rote-learning approach conducted using PAL ability, the orthographic approach conducted by PRA embodies the critical literacy skills needed by children midway through the process of learning to read. Hypothesis 2 was based on PA exerting a direct influence on PRA and an indirect influence through PAL. To further explore the effects of visual complexity on the supportive relation between PA and PAL, we divided PAL items into two conditions with different stroke numbers (HSPAL and LSPAL) for scoring, and then conducted two path analyses and proposed Hypothesis 3.

#### Hypothesis 1

The PRA but not the PAL of second-grade students is significantly associated with CS.

#### Hypothesis 2

The PAL of second-grade students partly mediates the association between PA and PRA.

#### Hypothesis 3

The PA of second-grade students is significantly associated with HSPAL, whereas their PAL is significantly associated with LSPAL.

## Method

### Participants

This study recruited 71 s-grade students from 2 summer daycare centers in Taiwan. It was originally conducted during the 2020 summer break before the participants entered the third grade. During the summer of 2022, we conducted a follow-up study to collect the general cognitive ability values of the same participants for inclusion as a control variable in the analysis model. Due to 1 participant not being accessible at this follow-up, the final sample for the hypothesized mediation model comprised 70 subjects. The mean age of the participants was 8.48 years (SD = 0.49 years), and 35 were male.

It should be noted that different Chinese scripts are used in different Chinese-speaking regions, with traditional Chinese characters currently used in Taiwan, Hong Kong, and Macao, and simplified characters being commonly used in mainland China and Singapore. Moreover, the Zhu-Yin-Fu-Hao phonological coding system is stipulated as compulsory learning content during the first 10 weeks of the first semester for first-grade students by the Ministry of Education in Taiwan.

The researchers asked teachers at the daycare centers to distribute consent forms to the students, and each student took their form home for their parents to sign if they agreed to their child participating in the study. Several days later, the researchers collected the consent forms from the daycare centers. The first author conducted tests at the daycare centers on predetermined dates. After the tests, the students were given stationery as a gift. The study was approved by the institutional review board of National Taiwan Normal University.

### Measures

#### General cognitive ability

To include general cognitive ability as a control variable in the hypothesized model, the present study employed a Chinese version of Raven’s Coloured Progressive Matrices-Parallel (CPM Parallel), which was revised by Chen and Chen (2006). CPM Parallel consists of 36 questions. Participants are asked to identify the missing item that completes a pattern from six options provided. CPM Parallel has a test–retest reliability of 0.73, a split-half reliability of more than 0.87, and a Cronbach’s *α* exceeding 0.85. The participants were rewarded 1 point for every correct answer, and so the maximum total score for the test was 36.

#### Phonetic radical awareness

PRA tests include phonetic consistency and regularity subtests developed by Tseng (2018). The PRA test aims to ascertain whether students can correctly recognize a phonetic radical and adopt the correct strategies to guess the pronunciation of a compound that they have not encountered previously. Participants were asked to choose the correct answer for each question from four options. Each question included a pseudocharacter, and participants were asked to guess how to pronounce the character. An example question is as follows: . There were seven questions pertaining to regularity and six to consistency. The student scored 1 point for each correctly answered question. Cronbach’s *α* values for the regularity and consistency questions were 0.75 and 0.82, respectively.

#### Phonological awareness

Li et al. (2012) and Siok and Fletcher (2001) found that onset–rhyme awareness (rather than phonemic and syllable awareness) predicted the Chinese reading ability of school-aged children, and this study therefore adopted the two subtests for onset and rhyme awareness from the PA test developed by Tseng et al. (2006). The test used fake sounds as stimuli. The listening material was played during the test, and students selected the phonetic notation that expressed the pronunciation that they heard. Each subtest consisted of eight questions, with each question worth 1 point, giving a maximum total score of 8 points for each subtest. Cronbach’s *α* values for the onset and rhyme awareness tests were 0.75 and 0.76, respectively.

#### Character size

One-character reading has been used by many researchers to assess Chinese reading ability (e. g., Georgiou et al., 2017; Li et al., 2012), and so this study adopted the A12 subtest of the one-character reading assessment developed by Hung et al. (2006). There were 40 characters in the test in total. During the test, students wrote down the phonetic notations of the characters and created a phrase using each character.

Two steps were used to convert the scores for the performance of these characters into CS: (1) the response for each test item was only considered correct if both the pronunciation and meaning of the character were correct, and (2) the number of correct responses was converted into CS using stratified sampling of the character frequency (Hung et al., 2006). This technique involves dividing all learned characters into multiple stratums and randomly selecting test items from each stratum. In scoring the responses of a student, the proportion of correct responses within each stratum is used to estimate the number of known characters, and total number of these characters across stratums is taken as the student’s CS. Both the internal consistency Cronbach’s α and split-half reliability coefficient exceeded 0.85, while the test–retest reliability coefficient exceeded 0.80.

### Paired–associate learning

#### PAL stimuli

Given that building print–meaning and print–sound mappings is a major goal of word acquisition (Perfetti & Hart, 2002; Seidenberg & McClelland, 1989), the present study included the visual–pronunciation PAL measured using a visual–verbal PAL task and the visual–semantic PAL measured using a visual–visual PAL task to evaluate the latent variable: the construct of PAL. The PAL stimulus materials were self-developed based on previous research (Chow, 2014; Georgiou et al., 2017; Li et al., 2009; Liu & Chung, 2021; Liu et al., 2021). The Appendix lists the 20 coined stroke patterns used and the corresponding syllables and figures. The method used to develop PAL material is described below.

For visual materials, instead of using glyphs of real radicals used in Chinese characters, we designed 20 novel coined patterns of strokes that intersected and connected in legitimate ways: 10 each for LSPAL and HSPAL. The mean numbers of strokes in the LSPAL and HSPAL conditions were 3.80 (SD = 0.92) and 8.50 (SD = 2.37), respectively. Each of these 20 coined stroke patterns had randomly paired pronunciations and semantics to form stimulus materials for visual–semantic and visual–pronunciation PAL, respectively.

The pronunciation information included 20 common Chinese character syllables, which were presented using the Zhu-Yin-Fu-Hao phonological coding system during the PAL task. Based on the data from the investigation report database of the characters and words used by elementary school students (National Languages Committee, 2002) and Chinese character orthography database (Chen et al., 2011), the mean log-transformed frequencies (per million) of Chinese character syllables for HSPAL and LSPAL were 3.49 and 3.14, respectively. A *t*-test revealed that syllable frequency did not differ significantly between the two sets of PAL tasks [*t*(18) = 1.589, *p* = 0.130].

The semantic information used concrete visual figures to reduce the impact of differences between the imaginations of the students that could potentially result from verbal input. The Appendix shows the semantic figures; the 20 figures for HSPAL and LSPAL were paired in the same semantic categories (e.g., plants, animals, objects, and actions). The figures belonged to semantic categories familiar to children and had novel features. Given the novelty features, we were mindful that the accessibility of the semantic figures could influence the PAL performance. After examining 20 semantic figures, 5 were identified as less accessible to children (i.e., numbers 5, 6, 9, 15, and 19 in the appendix) and thus deleted. We used *t*-tests to compare the accuracies between the 15 figures and the original 20 figures for visual–semantic HSPAL, visual–semantic LSPAL, visual–pronunciation HSPAL, and visual–pronunciation LSPAL, which revealed no significant differences: *F*[1,69] = 2.655 (*p* = 0.107), *F*[1, 69] = 1.849 (*p* = 0.178), *F*[1, 69] = 1.657 (*p* = 0.202), and *F*[1, 69] = 1.136 (*p* = 0.290), respectively. These results suggest that these 5 less accessible semantic figures did not cause notable interference to the PAL performance.

#### PAL procedure

**Fig. 2:**
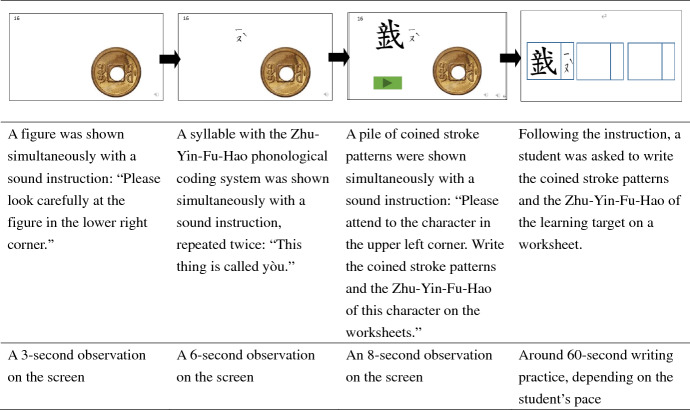
Example of the learning exercise with a worksheet

Three PAL learning exercise rounds were conducted once per week. During each week, students were asked to learn 20 pairs of PAL materials in random. The instructions for the task were as follows: “*In a moment you will see some strange objects that you have never seen before. Please remember how to pronounce the names of these objects and how to write them, and then write down each character and its phonetic notation twice on the worksheet.*” Each round typically took the students 10–15 min to complete. To learn each character, the students were asked to watch a PowerPoint presentation that sequentially displayed the semantic figures, syllables, and stroke patterns of the target character (Fig. 2). Next, the students were instructed to write the character and its phonetic notation for each target character.

As shown in Fig. 2, in the learning phase, each bit of information was presented step by step with animations and structuralized timeframe in the following sequence: the figure, syllable, and coined stroke patterns of a learning target. That is, the training procedure of presenting learning materials had clear instructions, guiding the learner’s attention to newly-presented information step by step. Although the procedures were different from traditional PAL tasks, which adopted independent training and testing, our design of training in the learning phase affords more authentic contexts and therefore may enhance the ecological validity of this study.

#### PAL posttests and scoring

**Fig. 3:**
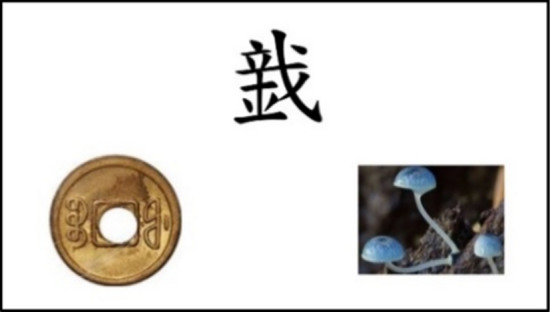
Example of the visual-semantic PAL test

**Fig. 4:**
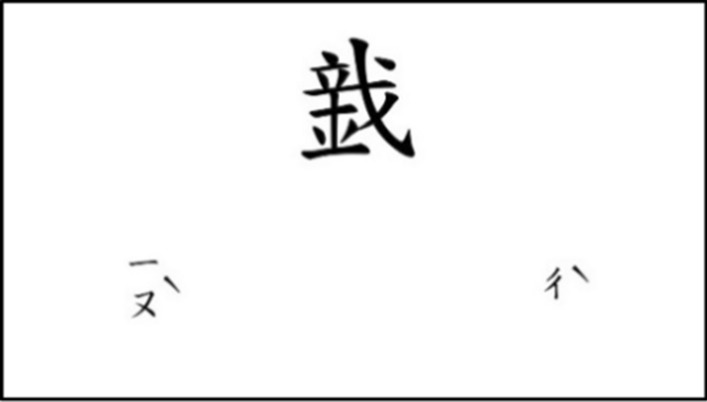
Example of the visual-pronunciation PAL test

The students underwent PAL posttests after the third round of PAL learning exercises. The PAL test was developed using PsychoPy Builder (version 2020.1.3), and conducted using computers. Participants completed the visual–semantic PAL test and then the visual–pronunciation PAL test, and had to choose the correct answer in each from two possibilities. In the visual–semantic PAL test (Fig. 3) the participant had to select the image to which the character in the middle of the figure referred, while in the visual–pronunciation PAL test (Fig. 4) the participant had to select the phonetic notation that indicated the pronunciation of the character. Each coined stroke pattern appeared twice, with the positions of the two images reversed between the two tests. To reduce the guessing rate, two questions that only differed in their position on the screen had to be answered correctly to be considered correct, and 1 point would then be awarded. Each of the visual–semantic HSPAL, visual–pronunciation HSPAL, visual–semantic LSPAL, and visual–pronunciation LSPAL conditions had a maximum score of 10 points.

The PAL posttests exhibited good construct validity. The four PAL conditions were positively correlated with the number of characters learned by the students (*r* = 0.25 ~ 0.62, *p* < 0.05). A two-way mixed-design variance analysis of the PAL test scores was performed on the different mapping types (visual–semantic and visual–pronunciation PAL) and different stroke numbers (HSPAL and LSPAL). Consistent with previous findings (Chen et al., 2008; Su & Samuels, 2010), the number of strokes significantly affected PAL performance, with the score being higher for LSPAL (mean = 6.34) than for HSPAL (mean = 5.89) (*F*[1,69] = 5.03, *p* < 0.05). The results also indicate that different mapping types and numbers of strokes did not exert an interactive effect on the score (*F*[1,69] = 1.51, *p* = 0.22); there was also no difference between the scores for different mapping types (*F*[1,69] = 0.02, *p* = 0.90). Regarding reliability, the coefficient for the correlation between the four PAL conditions ranged from 0.66 to 0.77.

### Procedure

The tests were conducted in small groups of two or three people, with each group guided by a trained undergraduate student. The tests were performed in classrooms once per week at a fixed time over three consecutive weeks: tests on basic information, PRA, PA, and the first round of PAL learning exercises during the first week; the CS test and the second round of PAL learning exercises during the second week; and the third round of PAL learning exercises and a PAL posttest during the third week.

### Statistical analysis

A general-case path analysis with the latent variables of a structural equation model (SEM) and maximum-likelihood estimation procedures were applied to the model using Amos software (version 26). The following statistical indicators of the goodness of fit of a model were selected based on the suggestion of Kline (2005): (1) chi square (*χ*^2^), (2) root-mean-square error of approximation (RMSEA), (3) comparative fit index (CFI), and (4) standardized root-mean-square residual (SRMR).

PA could exert direct and indirect effects on PRA, with the former assessed after controlling for effects from the mediator PAL. The indirect effects of PA on PRA through PAL were assessed using the bootstrapping technique of Preacher and Hayes (2008) to establish confidence intervals (CIs) to account for indirect effects (with 1000 iterations). The CI can be used to assess whether there is an indirect effect; that is, whether the inclusion of a specific mediator (PAL) significantly reduces the effect of PA on PRA.

## Results

Table 1 lists the Cronbach’s *α*, descriptive statistics, and Pearson’s *r* correlation coefficients for all of the variables. It is worth noting that their mean CS was 1205.19, while the nationwide survey by Wang et al. (2008) found that second-grade students had a mean CS of 1248, which supports the representativeness of the sample in the present study.

**Table 1:** Cronbach’ *α* values, descriptive statistics, and Pearson’s *r* correlation coefficients for the variables (*N* = 70)

Measure (maximum possible score)	Cronbach’s *α*	*Mean*	SD	2	3	4	5	6	7	8	9	10
1.General cognitive ability (36)	.85	28.44	5.04	.31**	.31**	.20	.31**	.32**	-.01	-.04	.42**	.35**
2. Character size (2126)	.91	1205.19	422.40	1	.47**	.32**	.43**	.30*	.36**	.24*	.58**	.57**
*PAL*												
3. Visual-semantic HSPAL (10)	.77	5.79	2.45		1	.39**	.68**	.32**	.22	.16	.21	.33**
4. Visual-pronunciation HSPAL (10)	.76	6.00	2.54			1	.32**	.44**	.36**	.09	.13	.36**
5. Visual-semantic LSPAL (10)	.74	6.49	2.43				1	.24*	.15	.12	.28*	.42**
6. Visual-pronunciation LSPAL (10)	.66	6.20	2.67					1	.06	-.06	.20	.25*
*PA*												
7. Onset awareness (8)	.75	6.37	1.53						1	.53**	.22	.34**
8. Rhyme awareness (8)	.76	6.59	1.40							1	.23	.30*
*PRA*												
9. Regularity (7)	.75	5.78	1.66								1	.60**
1. Consistency (6)	.82	3.93	1.55									1

*LSPAL* low-stroke-count PAL, *HSPA*L, high-stroke-count PAL, *PA* phonological awareness, *PRA* phonetic radical awareness, *PAL* paired association learning

*p < .05, **p < .01

### Goodness of fit of the model

**Fig. 5:**
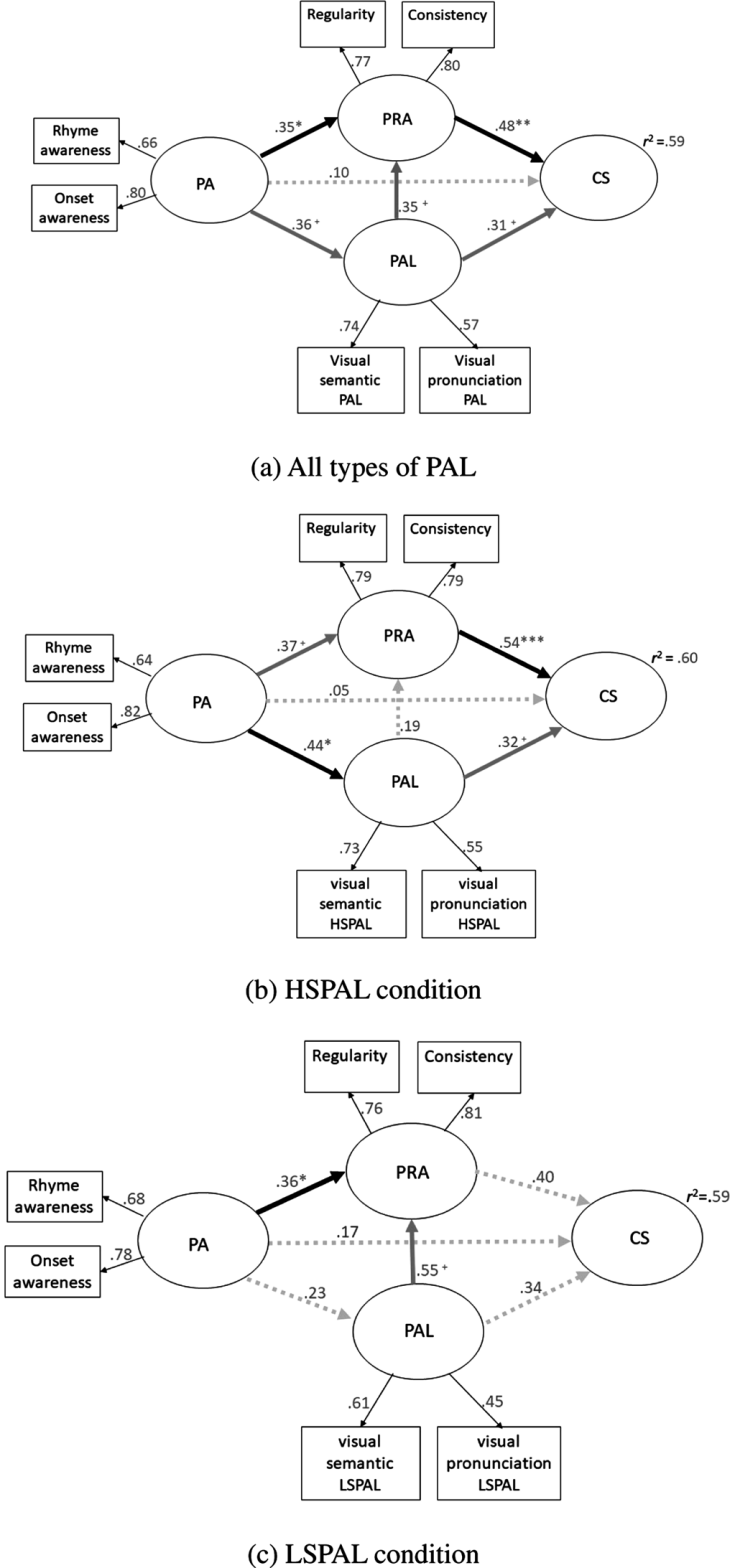
Standardized path models depicting the model for predicting CS using PA (*N* = 70). Note. General cognitive ability was included in the model as control variable but is not represented in these diagrams. Note. Solid black lines indicate significant paths, solid gray lines indicate marginally significant paths, and dotted gray lines indicate nonsignificant paths. + *p* < .10, *p < .05, **p < .01, ***p < .001. Note. CS, character size; PA, phonological awareness; PRA, phonetic radical awareness; PAL, paired association learning; LSPAL, low-stroke-count PAL; HSPAL, high-stroke-count PAL

The hypothesis model used the observed variables of PAL semantics and pronunciations to estimate the latent variable PAL, used regularity and consistency to estimate the latent variable PRA, and used the onset and rhyme awareness tests to estimate the latent variable PA. Meanwhile, the control variable general cognitive ability was significantly and positively correlated with PAL and PRA (*r* = 0.302 ~ 0.416, *p* < 0.05), but it was not significantly correlated with PA (*r* = –0.036 ~ 0.023, *p* > 0.05). PAL and PRA were therefore regressed by general cognitive ability in the model. The SEM results indicated that the chi-square distribution of the overall PAL model was not statistically significant (*χ*^2^ = 11.55, *df* = 13, *p* = 0.57), suggesting that there was no significant difference between the model assumption and the actual observation. CFI, RMSEA, and SRMR were 1.00, 0.00, and 0.04, respectively, which were within the ideal ranges (≥ 0.95, ≤ 0.08, and ≤ 0.08, respectively) and hence indicate that the hypothetical model had an acceptable goodness of fit. Figure 5a shows the hypothetical results of the SEM analysis. All of the factor loadings of the three latent variables exceeded 0.5, demonstrating that they had high explanatory power and good construct validity.

### Path analyses: direct and indirect effects

Figure 5 represents the standardized path models depicting CS predictions using PA. More specifically, Fig. 5a shows the PAL score calculations for all items, while Fig. 5b and c show the calculations for the LSPAL and HSPAL items, respectively. Figure 5a illustrates that PRA and PAL accounted for 59% of the CS variance, and that PRA had a significant independent effect on CS (*β* = 0.48, *p* < 0.01), while PAL had a marginally significant association with CS (*β* = 0.31, *p* = 0.08), which supports Hypothesis 1.

The results of this study also supported Hypothesis 2. Table 2 indicates that the indirect effect of PA on PRA was 0.16, and that the 95% CI for the bias-corrected percentile method did not include 0 (0.02 ~ 1.14, *p* = 0.06), suggesting that PAL played the mediating role in the relationship between PA and PRA, while the indirect effect was weak. Furthermore, the direct effect of PA on PRA was 0.16, and the 95% CI included 0 (–0.23 ~ 0.74, *p* = 0.39), indicating that PAL fully mediated the relationship between PA and CS. The total effect of PA on PRA was 0.36, and the 95% CI did not include 0 (0.09 ~ 0.89, *p* < 0.05).

**Table 2:** Total, direct, and indirect effects of PA on PRA through PAL (*N* = 70)

	Estimate	95% confidence interval		
		Lower	Upper	*p*
Indirect effect	.16	.02	1.14	.06
Direct effect	.19	−.23	.74	.39
Total effect	.36	.09	.89	.03

Finally, Fig. 5b shows that PA was significantly associated with HSPAL (*β* = 0.44, *p* < 0.05), and Fig. 5c shows that there was no association between PA and LSPAL (*β* = 0.23, *p* = 0.26), supporting Hypothesis 3*.*

## Discussion

This study examined how PAL and PA interact and contribute to PRA under the assumed developmental trajectory from the rote-learning to the orthographic approach. This study recruited 70 s-grade Chinese students to participate in various tests and PAL tasks over 3 weeks. The path analysis found that (1) PRA but not PAL was significantly linked to CS, (2) PAL partially mediated the association between PA and PRA, and (3) PA had a significantly positive effect on HSPAL but was not associated with LSPAL. Below we discuss the main results of this study alongside those of previous studies, speculate about differences between Chinese and alphabetic writing systems, and end with pedagogical implications, limitations, and conclusion.

### Rote-learning and orthographic approaches on CS

Our study was consistent with extensive previous studies (Ho & Bryant, 1997a; Ho et al., 2003a; Li et al., 2020, 2021; Yin & McBride, 2015) in finding that the PRAs of second-grade students significantly contributed to CS whereas PAL had no additive effect on CS, supporting Hypothesis 1. This suggests that Chinese children who develop literacy rapidly do not tend to build an arbitrary connection at the whole-character level (via the lexical route); instead, they primarily adopt PRA at the radical level (via the sublexical route) in a top-down manner. In terms of the overall developmental trajectory, our findings are consistent with the sight word theory proposed by Ehri (2005) and the self-teaching theory (Share, 1995) developed based on alphabetical languages, suggesting that the acquisition and application of OTP rules within a writing system are of universal importance in word acquisition.

Liu and Chung (2021) found that OA and PAL made unique contributions to word reading among 204 preschool children. The present study extended the participants to second-grade students, and after considering OA (PRA in this study) found only a marginally significant relationship between PAL and word reading. Meanwhile, our additional mediation analysis found that when considering mediating roles of PRA and PAL, PA no longer exerted a marginal direct effect on CS. In short, PAL may act as the main character recognition strategy only at the preschool stage. After learning to read, children have acquired basic orthographic knowledge and therefore they would switch to orthographic approach as the main learning strategy when they encounter new characters.

Our finding of PAL not having an additive effect on CS differs from data for a population of English monolinguals (Wang et al., 2017a, 2017b). That study found that visual–verbal PAL predicted word learning even after controlling for the effects of phonological decoding and orthographic knowledge as quantified by the ability to read irregular words. The differences between the present study and that of Wang et al., (2017a, 2017b) could be attributed to differences in dependent variables and in the orthographic depth of the languages. First, the present study used CS (a static measurement) as the dependent variable, whereas Wang et al., (2017a, 2017b) used learning performance on nonwords, which involves a dynamic process of acquiring new orthographic representations. PAL is a dynamic ability to learn pairings between new items, and hence might contribute more to learning nonwords. Second, the effect of OA on CS would increase with the orthographic depth of the text (McClung et al., 2012). Skilled readers of a language with deep orthography, such as Chinese, primarily employ a decoding strategy that utilizes both the constituent small and large units (Goswami et al., 2001).

### Association between PAL and PRA

This study unexpectedly found only a marginally significant association between PAL and PRA. The results indicated that PAL may play a partial role in constituting PRA. Multiple factors might contribute to PRA, which is conceptualized as crystallized knowledge (Wang et al., 2017a, 2017b). Therefore, the children in the present study might have derived OTP rules of the script based on their literacy experience by PAL through additional implicit learning ability or explicit teaching (Ehri, 2005). In Taiwan, most school teachers adopt a word-based language teaching model, and additional explicit PRA teaching might only be provided to students with learning difficulties. Alternatively, statistical learning is the ability to understand the relationships between language materials based on language learning experiences (Aslin et al., 1998; Saffran et al., 1996), which might be an important internal mechanism underlying PRA. He and Tong (2017) found that Chinese children develop their PRA via a statistical learning mechanism by implicitly detecting statistical relationships between phonographic and orthographic inputs.

### Direct and indirect effect (via PAL) of PA on PRA

Our path analyses through overall PAL (Fig. 5a) revealed a significant direct effect of PA on PAL, which was consistent with Liu and Chung (2021) showing that PA influenced word reading via PAL among kindergarteners. The present study has produced evidence of the robust integration of PA and PAL by finding a significant association between PA on PAL even when taking PRA into account. Ho and Bryant (1997a) found a significant positive correlation between PA and OPC rules (i.e., PRA) among first-grade students. The present study further included PAL and found that the association between PA and OPC rules was partially mediated by PAL. The findings not only support the results of Ho and Bryant (1997a, 1997b), by demonstrating that PA helps children to use the phonetic component in Chinese characters, but further elucidate the effect of PA on PRA by confirming the mediating role of PAL.

In PAL learning, PA establishes an accurate phonological representation and facilitates the process in distinguishing homophones, making the connections more robust. Thus, when students encounter new characters, successful PAL consolidates the connections among the form, sound, and meaning of the new characters stored in memory. Furthermore, the development of PRA involves mastering the OTP rules of the Chinese writing system. The accumulation of literacy experience (i.e., the connections between the form, sound, and meaning of characters) therefore becomes one of the foundations for the development of PRA.

This study found that PA did not exert a significant direct influence on OPC rules learning and/or assisted in applying the OPC rules to word reading among Chinese second-grade students. In contrast, Ehm et al. (2019) found that the PA of German children was not only directly related to PAL, but also directly related to letter(s)–sound correspondence rules that are the script–sound conversion knowledge from alphabetic script, such as PRA in Chinese. This difference between writing systems may be attributed to PA helping a reader of alphabetic script to decode letters into phonemes, thereby enhancing the acquisition of the letter(s)–sound correspondence rules. However, in Chinese, the phonetic radical holistically represents the sound rather than analytically in any phonetic segments, and may therefore require less PA (Ho & Bryant, 1997a, 1997b).

### PA supports the phonological representation of HSPAL more than that of LSPAL

This study also used a model to explore how children adapt to the visual complexity of Chinese characters. Increasing the number of strokes embedded in PAL strengthened the connection between PA and PAL. This indicates that increasing the visual complexity of novel characters may subsequently increase the dependence on PA.

Considering the close relationship between PAL function and working memory processing (Berninger et al., 2015; Wang & Allen, 2018; Wang et al., 2017a, 2017b), as well as the low and cross-modal shared attentional resources (Engle et al., 1999; Matusz et al., 2015) in children, our findings suggest that increasing the attention resource of storing new orthographic codes in memory—which is demanded by PAL—can increase the reliance on PA efficiency to maintain the quality of the phonographic code. This result was also consistent with the assumption that central attentional resources are shared across verbal and visuospatial working memories (Alloway et al., 2006). However, since the present study is the first to test this assumption, further research is needed to determine how PAL interacts with multicomponent working memory.

Our findings regarding PA can be summarized as follows: The results showing that PA was not related to PRA indicated that, in Chinese lexical processing, PA might not function on phonological recording via PRA. Although PRA helped the children to learn characters, PA did not appear to play a role in this process. In contrast, our findings underscored the direct role of PA in supporting the rote-learning approach conducted using PAL ability. Specifically, PA (1) strengthens the lexical connections among a large number of characters by establishing clear phonological representations, and (2) reduces the cognitive load inherent in the visual complexity of characters, so as to support the development of Chinese reading ability.

### Pedagogical implications

Our findings suggest that PA is a core skill to develop the ability of learning to read. Therefore, to leverage PA in supporting rote-learning approach, Chinese instruction for students with dyslexia should focus on providing pedagogical designs that support PAL function, such as strengthening Zhu-Yin-Fu-Hao learning or reducing visual complexity of learning targets. To support Zhu-Yin-Fu-Hao mastery, aside from rote memorization, teachers could provide additional familiar semantic contexts to promote sophisticated PA, such as familiar words containing the target phonological notation or keyword mnemonics (e.g., Ehri et al., 1984; Fulk et al., 1997; Tsai et al., 2021). At the same time, students should master common semantic radicals and their most frequent positions in advance, such as  (mù, wood) and  (kǒu, mouth), to reduce the visual complexity of glyphs.

On the other hand, cognitive difficulties among Chinese students with dyslexia are quite diverse, including in PA (Ho & Lai, 1999; Ho et al., 2000), PAL (Ho, 2014; Li et al., 2009), and PRA (Ho et al., 2006). The model examined in the present study found a development route of important cognitive abilities, which could provide reference data for relevant personnel to systematically diagnose and identify the source of cognitive deficits in individuals with dyslexia.

### Limitations

Several limitations of this study should be acknowledged. First, it had a cross-sectional design and used data from second-grade students in the path analysis. There may be a bidirectional correlation between CS and the two strategy skills (PAL and PRA) or among the three cognitive language skills (PA, PAL, and PRA), which would be an alternative explanation for the obtained results. To rule out this possibility, future studies with a longitudinal design should collect data from the preschool stage to validate the hypothesized model based on more-robust evidence. Second, we are mindful that vocabulary knowledge is an important control variable when constructing a model of vocabulary development. However, due to the restricted timeframe for collecting data during the COVID-19 pandemic, vocabulary knowledge was not included in the present research model. Third, although the logographic type chosen in our PAL task provides an important base for beginning learning, they are few such characters (e.g., 3% in the Kang Xi Dictionary; DeFrancis, 1989). Future studies should include other types of characters, such as compounds. Multiple resources of visual complexity (e.g., number of components and spatial layouts) could then be included in the manipulation of visual complexity to further investigate the relationship between visually complexity embedded in PAL and PA.

### Conclusion

This study aimed to determine the roles of PA and PAL in the acquisition of Chinese reading ability by setting developmental trajectory using two recognition approaches based on previous studies. Our path analyses revealed that PAL in second-grade children fully mediates the association between PA and PRA. This finding extends the existing literature by indicating that it is PAL supported by PA, rather than PA itself, that increases PRA acquisition. This study also found that PA supports the phonological representation of HSPAL more than that of LSPAL. This preliminary evidence indicates that sophisticated PA may be required to support PAL due to the low processing resources induced by the demanding visual orthographic coding of Chinese characters. In summary, while this study supports that PA and PRA are universally important cognitive abilities, their roles in the acquisition of reading ability might be influenced by variations in the orthographic depths of different writing systems.
